# Current Practice in Preoperative Virtual and Physical Simulation in Neurosurgery

**DOI:** 10.3390/bioengineering7010007

**Published:** 2020-01-03

**Authors:** Elisa Mussi, Federico Mussa, Chiara Santarelli, Mirko Scagnet, Francesca Uccheddu, Rocco Furferi, Yary Volpe, Lorenzo Genitori

**Affiliations:** 1 Department of Industrial Engineering, University of Florence, via di Santa Marta, 3, 50139 Firenze, Italy; chiara.santarelli@unifi.it (C.S.); francesca.uccheddu@unifi.it (F.U.); rocco.furferi@unifi.it (R.F.); yary.volpe@unifi.it (Y.V.); 2 Department of Pediatric Surgery, Meyer Children’s Hospital, Viale Pieraccini 24, 50141 Florence, Italy; federico.mussa@meyer.it (F.M.); lorenzo.genitori@meyer.it (L.G.)

**Keywords:** neurosurgery, brain, cancer, 3D printing, computer aided design, 3D casting, additive manufacturing, virtual planning, physical simulation, preoperative planning

## Abstract

In brain tumor surgery, an appropriate and careful surgical planning process is crucial for surgeons and can determine the success or failure of the surgery. A deep comprehension of spatial relationships between tumor borders and surrounding healthy tissues enables accurate surgical planning that leads to the identification of the optimal and patient-specific surgical strategy. A physical replica of the region of interest is a valuable aid for preoperative planning and simulation, allowing the physician to directly handle the patient’s anatomy and easily study the volumes involved in the surgery. In the literature, different anatomical models, produced with 3D technologies, are reported and several methodologies were proposed. Many of them share the idea that the employment of 3D printing technologies to produce anatomical models can be introduced into standard clinical practice since 3D printing is now considered to be a mature technology. Therefore, the main aim of the paper is to take into account the literature best practices and to describe the current workflow and methodology used to standardize the pre-operative virtual and physical simulation in neurosurgery. The main aim is also to introduce these practices and standards to neurosurgeons and clinical engineers interested in learning and implementing cost-effective in-house preoperative surgical planning processes. To assess the validity of the proposed scheme, four clinical cases of preoperative planning of brain cancer surgery are reported and discussed. Our preliminary results showed that the proposed methodology can be applied effectively in the neurosurgical clinical practice both in terms of affordability and in terms of simulation realism and efficacy.

## 1. Introduction

A brain tumor’s severity is mainly assessed by considering its grade and the originating tissue. Such an assessment is needed to provide the care team with an understanding of the tumor’s growth and how to match optimal treatments towards each individual [1]. Surgery is the most effective approach for the treatment of brain cancer, with the primary aim of performing, when possible, a complete resection. Brain surgery is a complex procedure that is not exempted from risks. In fact, a surgical error can cause the patient irreversible neurological deficit or even, in the worst case, their death. For this reason, surgeons spend a considerable amount of time, before entering the operating room, studying the patient’s imaging and accurately planning the intervention. Such a process, named “preoperative planning”, often determines the success or failure of the surgical procedure. In this context, modern advances in 3D technology have enabled the development of surgical simulators that provide a realistic representation of complex anatomies. These simulators can be used as an aid for planning complex interventions. More specifically, reverse engineering, CAD (Computer-aided design) modeling, and additive manufacturing technologies [2,3] enable the fabrication of patient specific and high-resolution simulators, which provide a realistic and immersive training environment [4]. An in-depth study of the clinical case through the customized simulator allows surgeons to reduce surgery times and complication rates by helping them to predict surgical crucial points, identify adapted surgical strategies, and improve surgical outcomes [5,6]. The improvement of computer capabilities and the availability of more cost-effective medical image processing software and of affordable 3D printers could empower clinicians with more flexibility to design and execute personalized therapeutic plans. This enables satisfying the specific clinical needs of individual patients with affordable costs and reduced time, thus facilitating the mass personalization of the treatments, even during the daily practices of neurosurgical departments. In particular, next generation 3D printers allow the perfect reproduction of internal anatomies that look, feel, and operate like real anatomies, without the need for further painting or assembly [7]. Such an approach is, however, not always applicable, mainly due to the need for complex models consisting of more than one material color and stiffness (e.g., involving the brain, skull, vasculature, or tumor). The need for professional-grade printers and medical and technological skilled experts is the principal limit preventing the everyday use of 3D biomodels in clinical settings. Indeed, the involvement of experts in all phases of the manufacturing process makes the production of a biomodel expensive. As an example of this, it is worth considering that employing a junior engineer costs 25–50 €/h. The definition of a workflow methodology which standardizes the design and production of preoperative models can reduce the involvement of skilled experts and the rate of production errors, leading in a reduction of production costs for healthcare systems.

Accordingly, the goal of this paper is to provide a methodology for applying 3D-based technology simulators (including virtual modeling, 3D printing, and casting) for aiding surgical planning of brain tumor interventions through an optimized repeatable process. In particular, we provide the reader with different fabrication approaches (i.e., materials and methods) using relatively inexpensive materials, commercial-grade printers, and open-source and freeware software with the aim of optimizing the costs and effectiveness of manufacturing. The fabrication strategy is determined based on the patient’s specific anatomy and the pre-surgical simulation goal. To explore the realism of the proposed methodology and evaluate its usefulness, we report four clinical cases that underwent neurosurgical treatment with the neurosurgery team of the Meyer Children’s Hospital in Florence. Our preliminary results showed that the proposed methodology can be employed efficiently in a neurosurgical clinical practice, ensuring increased affordability and simulation realism which in some cases enables surgeons to identify an alternative and minimally invasive surgical approach compared to the traditional one.

## 2. The Surgical Procedure and Planning

**Figure 1:**
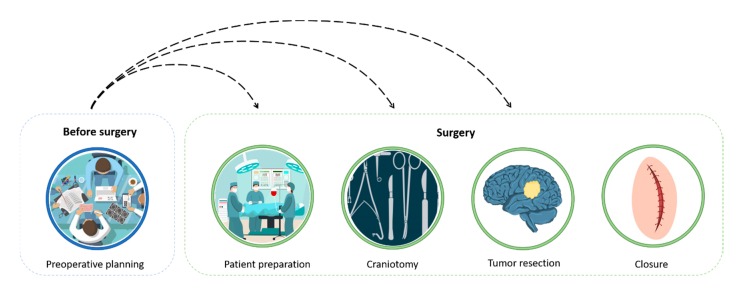
Brain surgery schematic pipeline. Preoperative planning drives the entire procedure.

As stated in the introductory section, brain cancer surgery is a complex procedure that is not risk-free, and possible complications can arise and even cause irreversible neurological deficits for the patient [8]. The entire surgical procedure involves several phases: preoperative planning, preparation of the patient and the operation area, craniotomy, tumor resection, and skull and scalp closure, as shown in Figure 1. Preoperative planning can heavily influence the entire process, since during the simulation, the neurosurgeons can decide on their entire surgical strategy, including how to position the patient, how to perform the craniotomy, and how to access to the tumor. For these reasons, the surgical planning is considered to be a critical step in many interventions [9]. Once the patient is taken to the operating room, after the anesthesia administration, his head is immobilized, and the operating area is drawn on the scalp (i.e., patient preparation). During the craniotomy phase, the surgeon creates an arched curl on the scalp overlying the lesion and the soft tissue is bent to expose the skull. A drill is then used to perform craniotomy, where a bone flap is removed and stored. Once the craniotomy is completed, the surgeon accesses the brain tumor according to the pre-planned trajectory. Often, the resection phase evolves entirely under an operating microscope [10] and partial or total brain cancer is removed. At the end of the procedure, during the closure phase, the removed bone flap is repositioned on the skull, is fixed with titanium plates and screws, then it is sutured.

### 2.1. CT/MRI-Based Presurgical Planning

Before surgery, the time the surgeons devote a preoperative plan is essential and often determines the level of confidence they have during the intervention. During this step, the surgeon defines the surgical problem, first to fully identify all the anatomical and technical aspects of the procedure, and then to plan the approach. The first aim of the planning is to establish a correct diagnosis that starts from the visualization of multimodal data obtained from various imaging modalities. Such imaging modalities can be functional or structural such as magnetic resonance imaging (MRI), computed tomography (CT), functional magnetic resonance imaging (MRI) [11], computed single photon emission tomography (SPECT), and so on. Based on this information, the surgeon, that has a thorough knowledge of the relevant operative procedures and the related hazards and success rates, makes his decisions on how to manage the intervention [12,13].

Preoperative planning is traditionally based on the identification of relevant anatomical reference points on tomographic data, on the measurement of the distance and angles between them, on the calculation of anatomical areas and volumes, and on the sketch of the possible trajectories for obtaining access to the tumor site. This process helps the surgeon to develop a correct 3D image of the problem in order to get a proprioceptive feel for the dynamics and complexity of the intervention to perform. Information on a surgical plan will ensure that the operating room staff has time to prepare for their patient and to identify and remedy potential difficulties [10]. The surgeon’s experience is paramount for achieving adequate outcomes. Notably, to avoid violating functional areas, and even after careful preoperative planning, the surgeon could prefer to perform a conservative surgery rather than an effective resection of the tumor. Such a limitation can be overcome by providing the surgeon with a tool that is able to (i) accurately and objectively predict the risk of a complete tumor resection, and (ii) provide a hands on experience of the surgery before entering the operating room.

### 2.2. Virtual and Physical 3D Presurgical Planning

An accurate and a high-resolution 3D reconstruction of the patient’s specific anatomy represents a major asset to the preoperative planning process. In neurosurgery, the development of virtual (VS) and physical simulators (PS) has allowed us to overcome some limitations of the traditional preoperative planning method (“in the mind” of the surgeon) [14]. Virtual simulators reproduce the surgical environments or settings including patient-specific anatomy, thereby allowing the surgeon to interact with these variables [15]. Nevertheless, the vision of the anatomy on a flat 2D computer screen makes often interpretations related to depth difficult and the cost of the simulator is high.

In contrast, physical simulators can be directly palpated, easily modified according to the physician’s request, incised by real surgical instruments, and manipulated.

Therefore, by using both physical and virtual simulators, it is possible to overcome the limits of traditional surgical planning, allowing the surgeon to understand the spatial relationships between vital structures and surgical targets. This in turn enables the surgeon to simulate different scenarios. In fact, the combination of virtual and physical simulation enables the identification of a set of promising surgical procedures that can be objectively compared to find the best intervention strategy. In addition, it makes the necessary clinical practices easier and safer for the treatment of patients, with the possibility for the clinicians to try procedures several times. In the state of the art research, there are some works dealing with the combination of virtual and physical simulators into a single mixed reality system, linking benefits coming from having a physical scenario to interact with and the potentialities offered by virtual reality [16]. More specifically, the combination of VS and PS: (i) provides objective and repeated measurements to help evaluate performance; (ii) allows us to easily change the anatomy, offering residents the possibility to try surgery not strictly based on a single example of anatomy; (iii) allows actual interactions with the simulated anatomy; and (iv) permits us to perform specific tasks with actual feedback. Among other things, VS and PS, allow for more precise and minimally invasive approaches, thereby reducing potential injury, eliminating the risks of serious complications, and thus improving the experience and manual skills of the operator, even in the management of possible situations of stress and error under crisis conditions.

**Figure 2:**
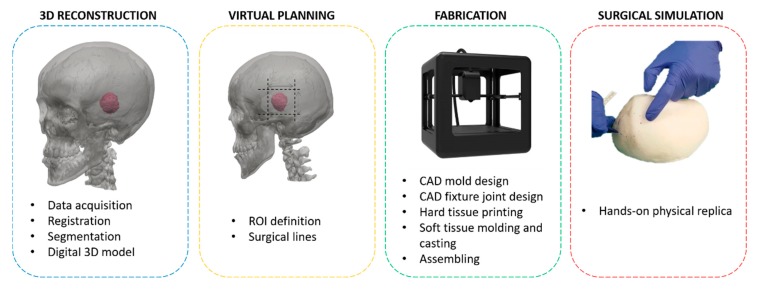
Simulation process pipeline.

As mentioned in the introductory section, the present work provides a method for applying 3D technology for realistic surgical simulator fabrications for brain tumor treatment which integrate virtual and physical simulations. In particular, the *simulation process* developed in this work consists of the following phases (see Figure 2):

(1) 3D reconstruction of the patient’s anatomy;
(2) Surgery virtual planning;
(3) Fabrication of the bio-model;
(4) Surgery simulation (the surgeon uses a hands-on bio-model to simulate the surgery).

While the 3D reconstruction is widely known, we focus on phases 2 and 3 in order to define a strategy which make the surgical procedure less invasive and provides a more realistic physical simulation.

The remainder of the paper is as follows: in Section 3 the method adopted to devise the virtual and physical simulators is described, while in Section 4 we report the case studies that have been carried out to prove the effectiveness of the combined use of virtual and physical simulation in the pre-operative planning of complex cases.

## 3. Simulation Process

In the following subsections, the various phases of the simulation process are described in detail.

### 3.1. 3D Reconstruction

The complete simulator manufacturing process includes some key steps represented in Figure 2 that, starting from 3D reconstruction of the patient anatomy, leads to the creation of 3D anatomical physical replica.

An effective simulator for preoperative planning and simulation requires an exact patient specific geometry 3D reconstruction. The first step consists of the acquisition of patient medical images (i.e., (CT/MRI)) that provide anatomical information. More specifically, MRI better represents many soft tissues, thus enabling an accurate segmentation and 3D reconstruction of tissues such as the brain and blood vessels. Instead, the reconstruction of bones is performed from the segmentation of a CT scan, which provides a clear variation between calcium-based tissues and other types of tissues in gray values. Since information gained from different images acquired in the clinical track of events is usually of a complementary nature, a proper integration is often needed. The first step of the integration process is to find the spatial transformation that best aligns different datasets, a procedure referred to as registration [17]. Such registration can be performed both in the 2D domain and in the 3D domain (i.e., after the reconstruction). The former requires the manual or automatic identification of several significant anatomical landmarks on both the considered modalities (usually CT scan and MRI scan). These are elaborated upon by specific image processing algorithms (i.e., mutual-information based [18] or Deep Neural Network based [19] algorithms), to determine the spatial roto-translation transformation needed to move the reference dataset into the coordinate system of the target dataset [20]. Registration in the 3D domain consists of the reconstruction of 3D models of anatomical structures from medical imaging and of the subsequent identification of significant 3D landmarks (in both the reference 3D dataset and in the target 3D dataset) As a result, by means of specific 3D processing algorithms (e.g., Iterative Closest Point [21] or Global registration algorithm [22]), the same spatial roto-translation transformation as in the image domain approach can be used [23]. The development of 3D models, for neurosurgical purposes, involves the image segmentation [24,25], of both soft tissues (e.g., brain, tumor, etc.) and hard tissues (e.g., skull bones). Registration and segmentation can be carried out with both commercial software (e.g., Mimics^®^, Amira^®^, etc.), which has a price range of 4000 € to 6000 €, and with open source software (e.g., 3D Slicer, OsiriX, ITK-SNAP, TurtleSeg, etc.). Within these software packages, once the segmentation of the anatomical element has been performed, the volume of the anatomical element is automatically obtained through embedded procedures, resulting in a three-dimensional configuration that can be stored in a 3D mesh file (i.e., an STL file format).

In order to contain costs and to focus the simulator on an optimized head replica, a region of interest (ROI) can be first defined to specify the surgical interest 3D boundaries. Such an operation can be performed, using the surgeon’s instructions in a commercial 3D modeling software environment (i.e., Fusion 360^®^, Geomagic Design XTM, Geomagic Freeform, Materialise 3-Matic, etc., whose prices range from 12,000 € to 18,000 €) or open source (i.e., Meshmixer, Blender). Such a process is needed to focus the simulation on a specific anatomical region, but could also be repeated to define the surgical lines needed to simulate the cranial resection process, thus leading the design to a partially operated simulator. 

### 3.2. Virtual Planning

Once the 3D model is reconstructed, it is possible to perform a virtual planning of the surgical intervention. With reference to neurosurgery, the main aim is to virtually determine the best surgical approach needed to access to the region of interest (i.e., the area where the tumor is located). In other words, the surgeon has to decide the position and orientation of a series of cutting planes to remove the bone and to easily reach the intervention area. The virtual simulation is carried out thanks to minimal cooperation between the surgeon and the CAD engineer.

In detail, by using an information-sharing platform, the surgeon can autonomously manipulate 3D models and provide engineers with all the design constraints needed for the resection planes. In particular, the 3D modeler Forger^®^, a mobile-based polygonal modeler running with an iOS operative system, demonstrated its effectiveness in dealing with virtual surgical planning [26]. 

With an intuitive touch screen interface, the surgeon can quickly pan, zoom, and rotate the 3D models and can easily add resection planes to the virtual model. Once the planes are correctly positioned on the model, it is possible to simulate the surgery by cutting the bone and soft tissues defined by the planes themselves (i.e., surrounding the tumor area). More different surgical strategies can be simulated using such a tool, to define the best approach (i.e., the minimally invasive one) to be further tested with the physical simulator. Moreover, the availability of the virtual model before and after the surgical simulation, allows us to manufacture the replica with and/or without the removed bone flap.

### 3.3. Fabrication

A rigid simulator part is certainly appropriate to reproduce the bone tissue but also, when needed, to reproduce a rigid replica of the soft tissues. The latter is preferred when neurosurgeon does not need to interact with the simulator through real surgical instruments, but instead needs to simply train the proprioception and observe the spatial relationships between the anatomical elements. When the simulation requires distinguishing soft tissues from rigid tissues, the soft parts are fabricated by casting silicon materials in rigid shell molds, thus following the same fabrication process of other rigid parts.

Once the virtual simulation has been carried out and the region of interest has been defined, the physical replica can be manufactured. This phase is divided into two different steps: rigid part fabrication and soft tissues fabrication.

#### 3.3.1. Rigid Parts Fabrication

The manufacturing process of rigid parts can effectively be performed with low cost 3D printer (available starting from about 300 €). Materials like PLA (Polylactic Acid) or ABS (Acrylonitrile Butadiene Styrene) [27] are commonly used as cost-effective materials to reproduce hard tissues like bones, thanks to their optimal properties such as model infill.

Following negative feedback from some surgeons, wood-loaded PLA (a material available on the market) was also tested to manufacture skull replicas. When PLA and ABS interact with the surgical drill, swarfs can be created adhering to the surface of the instrument, kneading it, and not allowing a correct and effective simulation of the cut. Wood-loaded PLA may represent a good choice to overcome this limit. In this case, the chip appears to be easily removable from the area of the tool, allowing us to easily perform the entire cutting procedure of the simulator.

The separated structures need to be equipped by fixture joints to be assembled with accuracy after printing. CAD tools are employed to insert the anchoring systems between the various anatomical elements present in the ROI through the insertion of pins or other engineering/mechanical solutions. The 3D model of the parts prior to being printed, must be first processed and optimized by setting some printing parameters [28], which can be the same for each simulator sharing similar design requirements.

The last step of the bio-model fabrication consists of the post-processing of the 3D printing output. When the prototype presents a poor surface finish, high porosity, the presence of appendices, supporting materials, and unfinished surfaces, an improvement can be obtained through a sandblasting process. The next step can involve the application of a resin coating to finish the surface of the final product. The resin is brushed on the object to fill all the model’s cavities or indentations and to improve surface quality, thereby smoothing the roughness and reducing the stair stepping effect typical of a 3D printed object [29,30].

#### 3.3.2. Soft Tissues Fabrication

The low-cost soft tissues fabrication process combines 3D printing and some tissue-mimicking material casting. Soft tissue structures can be obtained by casting silicone rubbers in 3D printed molds. Indeed, the ultra-soft nature of some human tissues, such as the brain, can be replicated thanks to silicone rubber with different hardness ranging from the ultra-soft scale (shore 00 scale) to the soft scale (shore A scale). Other materials such as alginate, agarose, poly (vinyl alcohol) (PVA), phytagel (PHY), Poly (ethylene glycol) (PEG), and polyurethanes are widely used in the literature [31], as their mechanical properties can mimic the elastic and haptic properties of human tissues. The fabrication process involves developing a mold design and engineering using CAD software. The mold is a negative replica of the anatomy, and it is fabricated with a 3D printing technique. The surfaces of release agents are then covered using an aerosol spray to prevent silicone rubbers from sticking to the mold, impeding the removal of the physical replica from the mold. When the silicone is being mixed with the catalyst, colorants and silicone additives can be added to the mixture, in order to modify the chromatic and mechanical properties of the rubbers, such as the realism and the tactile performance of the final product. To improve the outcomes of the simulator fabrication process, a vacuum degassing system can be used to degas the mixture before pouring [32]. The mixture is then poured inside the mold and after the polymerization time, and then the silicone replica is removed from the mold. Ultra-soft silicone rubbers have sticky and oily characteristics, making them not easily manageable. For this reason, a post processing of the physical replica is needed, and it consists of sprinkling talcum powder on the replica, eliminating the sticky effect. The last step is to assemble the individual parts and may require the use of glues to keep the individual elements fixed.

## 4. Case Studies

During this work, four case studies were carried out, which involved the fabrication of preoperative simulators for neurosurgical interventions involving the resection of meningioma. Written informed consent was obtained from the four patients, including for publication of both subjects’ data and all accompanying images. All methods were carried out in accordance with the guidelines laid down in the Declaration of Helsinki.

### 4.1. Case 1

**Figure 3:**
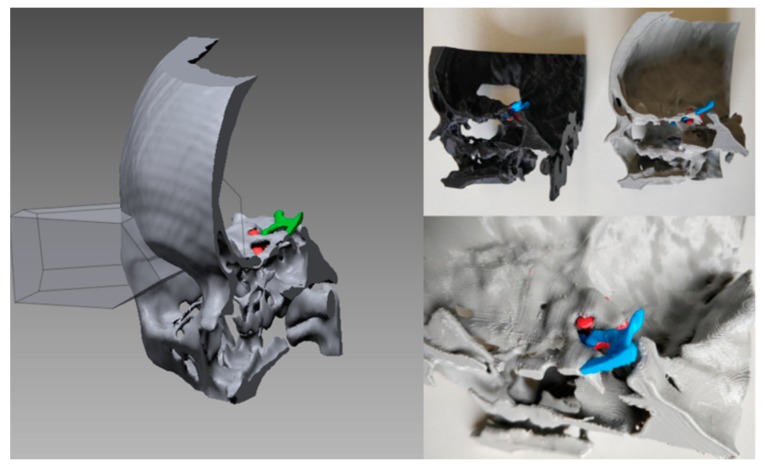
On the left, identification of cutting plans for access to the tumor; on the right biomodels fabricated in Polylactic Acid (PLA). The black model was manufactured directly with the cut identified by the surgeon at the virtual level. The grey model reproduces the entire anatomical portion involved in the surgery.

The first clinical case involved a sixteen-year-old girl suffering from a benign tumor at the base of the skull. The tumor was slightly compressing the optic nerve, thus making to accessing the tumor intracerebrally a hazardous process. The simulation objective was the identification of an alternative optimal surgical access option to preserve the optic nerve integrity. CT images (scanned with Philips Brilliance 64 machine; image size 512 × 512 px; xy spatial resolution 0.48 mm; slice spacing 0.40 mm) and MRI images (taken with Philips Medical Systems; image size 512 × 512 px; xy spatial resolution 0.53 mm; slice spacing 1 mm) were acquired and saved in a DICOM (Digital Imaging and COmunications in Medicine) format. After obtaining the 3D reconstruction with Materialise Mimics, using first phase of the pipeline shown in Figure 2, the STL file was imported into Geomagic Design X™ (3D Systems, Inc., Rock Hill, SC, USA) to identify the region of interest together with the neurosurgeon’s team. At a virtual level, several surgical lines were tested to arrive at the best strategy of intervention (Figure 3), which allowed us to preserve the primary brain areas and the optic nerve. Two fully rigid 3D models were manufactured with a 3D printer, one characterized by the entire anatomical portion involved in the intervention, the other instead faithful to the cuts identified at the virtual level. Both models were printed with MakerBot Replicator 2 (MakerBot, Brooklyn, NY, USA) with a Polylactic Acid (PLA) filament and are shown in Figure 3. The production time of the simulator was: four hours for 3D reconstruction, one hour for virtual planning, and five hours for 3D printing. The material price for 3D printing of each simulator was 2.60 €.

In similar clinical cases, the pterional craniotomy is the traditional approach for accessing the tumor through the brain by opening the dura mater. Thanks to careful preoperative planning, a neurosurgeon has been able to identify a transorbital route for the resection of the tumor that enabled the removal of the bone of the orbit and provided access to the tumor, making the surgery minimally invasive and providing a low risk of brain damage.

### 4.2. Case 2

**Figure 4:**
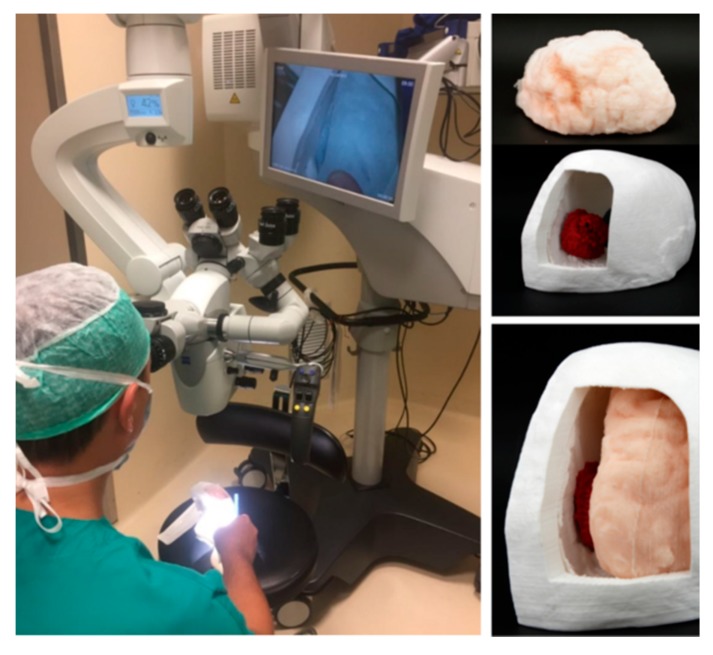
A biomodel which consists of a skull, brain, and tumor growth. A neurosurgical microscope was used to observe the spatial relationships between the anatomical elements involved in the surgical procedure with the biomodel.

This case study focused on the fabrication of a simulator for the intervention of a 67 years old patient with a meningioma at the tentorium level. The position of the tumor required an accurate study of the geometry and spatial location of the meningioma placed near a venous sinus. The anatomical parts involved in the construction of the simulator were the skull, the tumor, the brain, the tentorium, and the falx. CT images (scanned with a Philips Brilliance 64 machine; image size 512 × 512 px; xy spatial resolution 0.48 mm; slice spacing 0.40 mm) and MRI images (taken with Philips_Healthcare/Ingenia; image size 256 × 256 px; xy spatial resolution 0.93 mm; slice spacing 1 mm) were acquired and saved in a DICOM format. Starting from the reconstructed digital 3D model obtained from CT segmentation with Materialise Mimics, the physician indicated that a cut on the skull was needed to access the tumor area. Figure 4 shows the simulator that consisted of a replica of a skull, brain, and tumor. The skull and tumor were manufactured directly in PLA, while the brain was made in a super soft silicone rubber with a shore hardness of 00–50 (Ecoflex 00-50, Smooth-On, PA, USA) to replicate the mechanical characteristic of the actual human tissue. The manufacturing process of the soft tissue took longer than the direct 3D printing procedure for the skull and tumor. In fact, the negative of the brain was printed in FDM, while a resin coating (XTC-3D, Smooth-On, PA, USA) was brushed on the surface of the mold to eliminate the stair stepping effect. After the cure time (4 h), the silicone rubber was poured into the mold to obtain a positive anatomical replica. The production time of the simulator was: six hours for 3D reconstruction, one hour for virtual planning, eight hours for 3D printing, and five h for silicone parts fabrication. In this case, the material price of the 3D printed parts and mold was 12.00 € and the price of the silicone rubber parts was 17.00 €.

The models have been studied with the neurosurgical microscope to simulate the surgical approach with the aim of understanding the volumes, depth and spatial relationships between the tumor, brain, and tentorium in detail (Figure 4). Thanks to the use of simulators, it was possible to perform the operation in an optimized way by removing all the tumor tissue without affecting other anatomical areas.

### 4.3. Case 3

**Figure 5:**
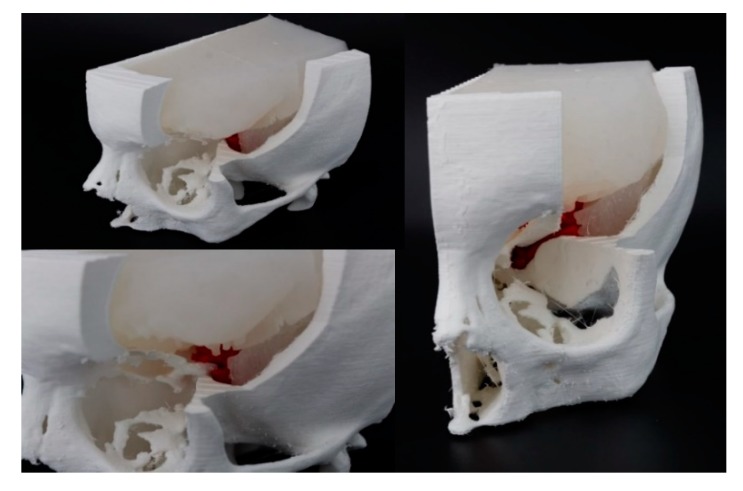
Different views of the biomodel that consisted of a skull, brain, and tumor.

The third simulator involved planning the removal of a meningioma during the clinoid process in a 55 years old patient. The simulator’s aim was to increase the neurosurgeon awareness of the spatial relationships between the patient’s brain, the cancer, and the big intracranial vessels. CT images (scanned with Siemens /Somaton Definition As+ machine; image size 512 × 512 px; xy spatial resolution 0.47 mm; slice spacing 1 mm) and MRI images (taken with Siemens/Aera image size 256 × 256 px; xy spatial resolution 0.98 mm; slice spacing 1 mm) were saved in a DICOM format. A different view of the simulator is shown in Figure 5, which consisted of a skull, brain, and tumor. In particular, the skull and tumor were manufactured in FDM while for the brain, the silicone Eco-flex 00-50 was used. At the digital level, the access cut to the tumor was identified so that the surgical route did not cross primary areas of the brain, thus reducing the risk of neuromotor and sensorineural deficits. The production time of the simulator was: four hours for 3D reconstruction, 1.5 h for virtual planning, six hours for 3D printing, and five hours for silicone parts fabrication. The CT segmentation was executed fully manually by an expert engineer supervised by the surgeon and radiologist, because of the low contrast of the tissue boundaries that needed to be reconstructed. In this case, the material price of 3D printed parts and mold was 9.00 € and the price of the silicone rubber parts was 10.00 €.

### 4.4. Case 4

**Figure 6:**
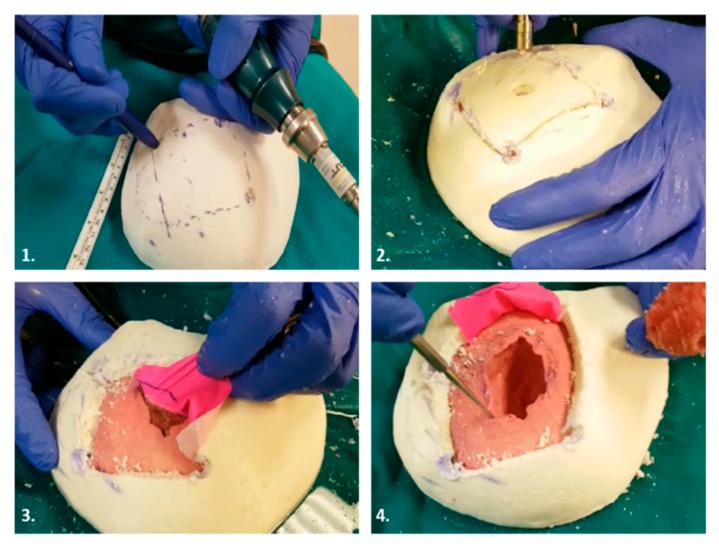
Simulation of the surgical procedure. **1**. Skull flap tracking with surgical skin marker pens; **2**. Craniotomy; **3**. Separation of meninges from meningioma tumors; **4**. Meningioma removal.

This case study concerned the manufacturing of a simulator used in the case of a 76 years old patient with a meningioma located in the temporal lobe closely related to the meninges. The surgical operation involved separating the meninges from the tumor mass without affecting any tissue involved in the operation. In this case, therefore, the simulator consisted of four parts: a skull printed in PLA loaded with wood fiber, a brain, a meningioma made with different silicones as tissues with different hardness levels (the first with Eco-flex 00-50 and the second with Dragonskin 10), and meninges reproduced with a thin rubber sheet. In this case, the cut was made physically on the simulator by the surgeon, using real surgical instruments. The simulator allows resident physicians to practice craniotomy centered on the lesion and therefore primarily has an educational purpose. Figure 6 shows the procedure that the surgeon performed on the physical model to simulate the entire surgery. In the first phase the possible access cuts were traced, the best cut was obtained by creating an opening using the craniotomy drill, and after separating the layer of latex from the brain and the tumor, the latter was removed. The production time of the simulator was: five hours for 3D reconstruction, one hour for virtual planning, six hours for 3D printing, and five hours for silicone parts fabrication. In this case, the material price of 3D printed parts and mold was 11.00 €, the price of the silicone rubber parts was 15.00 €, and the price of the thin rubber sheet was 1.70 €.

## 5. Costs and Timings Analysis

In the present section, an assessment of production times and costs is reported for applying the proposed method. Overall, the simulation process with the five phases described above requires about one week from the acquisition of diagnostic images to the actual surgery. The duration of each phase is closely linked to the simulator produced (volume, type, etc.) and to the complexity of the surgery. Therefore, it is not possible to define a precise time for each phase, but an average time range could be evaluated based on our experience. Table 1 shows the average times and the professional figures involved in each phase.

The production costs of 3D printed parts can be evaluated according to the following equation [33]:

(1) $$C_{p}=C_{e}+C_{m}+C_{t}$$

where $C_{e}$ is the production cost, $C_{m}$ is the cost of the 3D printed material, and $C_{t}$ is the processing cost of the 3D model and labor. (Table 2)

When the simulators are composed of both soft and rigid parts, the material costs of the silicone rubbers and additives must be added (see Equation (2), Table 3).

(2) $$C_{rs}=C_{p}+C_{sm}$$

where $C_{p}$ is the cost of 3D printed parts and $C_{sm}$ is the cost of the material for the soft parts.

## 6. Conclusions

Preoperative planning is a crucial step for any entire surgical procedure. A deep knowledge of the anatomical geometry of the patient is necessary to plan the best surgical strategy and to reduce the risk of surgical errors. Within this context, it is clear how important it is to use surgical simulators that allow the physician to simulate the operation before entering the surgical room, improving self-confidence and reducing stress during actual surgery [34]. Specifically, during this phase, the surgeon can test multiple surgical strategies in order to identify the best one in terms of results and timing. We have introduced a general pipeline to produce a low-cost 3D patient-specific anatomical model that would allow surgeons to conduct an accurate and successful preoperative planning process. The pipeline is the result of an analysis of literature on the use of 3D printing and tissue mimicking materials for education and surgical planning, and aims to introduce neurosurgeons and clinical engineers to a cost-effective and in-house preoperative simulation. The pipeline considers different fabrication methods that can be used based on the challenge and goal of preoperative planning, but are also strictly dependent on the specific pathological case. Different manufacturing choices can be made, but one of our objectives was to minimize production costs to make preoperative simulator usage more accessible and more common in clinical practice. We have reported our experiences with the neurosurgery team of Florence Meyer Children’s Hospital, focusing on four 3D printed models that were used to plan surgeries by following the pipeline proposed. The simulators produced allowed the surgeon to identify, in some cases, alternative surgical routes to the traditional ones, thus avoiding the risk of neurological deficits caused by a possible injury of healthy tissue. In addition, better surgical results have been obtained thanks to an in-depth knowledge of the volumes of diseased tissues and spatial relationships with other anatomical regions. In conclusion, the presented methodology aims to provide a clear and systematic procedure to make the creation of physical simulators in the clinical practice more affordable to provide benefits both for residents’ education and for senior surgeons involved in preoperative planning and simulation. Objective measurement of the surgical performance improvements requires a long-term observation of the clinical outcomes in a clinical trial. Using this perspective, future work will focus on the collection of quantitative experimental results on the improvement of surgeon performances using a simulator produced according to our methodology.

## Figures and Tables

**Table 1:** Timing data of the simulation process.

Phase	Day	Working Hours	Professional Figures
3D reconstruction	1	4–8	Radiologist, Engineer
Virtual planning	2	1–5	Engineer, Surgeon
Fabrication	2–3	2–16	Technician
Simulation	4	1–3	Surgeon
Surgery	5	4–8	Surgeon

**Table 2:** Costs of rigid parts for the manufacturing process.

Costs Analysis for the Prototyping of Items in A 3D Printer	
**Machine Depreciation Data $C_{e}$**	
Price of Machine (€)	2500
Yearly maintenance cost (€)	250
Years of depreciation	4
**Cost of Material Data $C_{m}$**	
Cost of material: ABS filament (€/kg)	~20
Cost of material: PLA filament (€/kg)	~22
Cost of material: wood-loaded PLA filament (€/kg)	~30
**Cost of Technical Analysis Data $C_{t}$**	
Cost of technical model analysis (€/h)	20

**Table 3:** The material costs for the production of soft tissues.

Material	Cost
Silicone Rubbers	~35 €/kg
Release Agents	~12 € one-time fee
Colorants	~15 € one-time fee
Silicone additives	~ 40 €/kg
3D printing coating	~25 € one-time fee

## References

[1] Rorke L.B., Gilles F.H., Davis R.L., Becker L.E. (1985). Revision of the world health organization classification of brain tumors for childhood brain tumors. Cancer.

[2] Guo N., Leu M.C. (2013). Additive manufacturing: Technology, applications and research needs. Front. Mech. Eng..

[3] Ngo T.D., Kashani A., Imbalzano G., Nguyen K.T.Q., Hui D. (2018). Additive manufacturing (3D printing): A review of materials, methods, applications and challenges. Compos. Part B Eng..

[4] Tai B.L., Rooney D., Stephenson F., Liao P.S., Sagher O., Shih A.J., Savastano L.E. (2015). Development of a 3D-printed external ventricular drain placement simulator: Technical note. J. Neurosurg..

[5] Oishi M., Fukuda M., Yajima N., Yoshida K., Takahashi M., Hiraishi T., Takao T., Saito A., Fujii Y. (2013). Interactive presurgical simulation applying advanced 3D imaging and modeling techniques for skull base and deep tumors: Clinical article. J. Neurosurg..

[6] Waran V., Narayanan V., Karuppiah R., Owen S.L.F., Aziz T. (2014). Utility of multimaterial 3D printers in creating models with pathological entities to enhance the training experience of neurosurgeons: Technical note. J. Neurosurg..

[7] Tejo-Otero A., Buj-Corral I., Fenollosa-Artés F. (2019). 3D Printing in Medicine for Preoperative Surgical Planning: A Review. Ann. Biomed. Eng..

[8] Mukherjee S., Pringle C., Crocker M. (2014). A nine-year review of medicolegal claims in neurosurgery. Ann. R. Coll. Surg. Engl..

[9] Nocerino E., Remondino F., Uccheddu F., Gallo M., Gerosa G. (2016). 3D Modelling and rapid prototyping for cardiovascular surgical planning-Two case studies. International Archives of the Photogrammetry, Remote Sensing and Spatial Information Sciences-ISPRS Archives.

[10] Ploch C.C., Mansi C.S.S.A., Jayamohan J., Kuhl E. (2016). Using 3D printing to create personalized Brain models for neurosurgical training and preoperative planning. World Neurosurg..

[11] Ottlakan A., Borda B., Morvay Z., Maraz A., Furak J. (2017). The effect of diagnostic imaging on surgical treatment planning in diseases of the thymus. Contrast Media Mol. Imaging.

[12] Grau S., Kellermann S., Faust M., Perrech M., Beutner D., Drzezga A., Zöller J. (2018). Repair of cerebrospinal fluid leakage using a transfrontal, radial adipofascial flap: An individual approach supported by Three-dimensional printing for surgical planning. World Neurosurg..

[13] Hoang D., Perrault D., Stevanovic M., Ghiassi A. (2016). Today surgical applications of three-dimensional printing: A review of the current literature & how to get started. Ann. Transl. Med..

[14] Ferroli P., Tringali G., Acerbi F., Schiariti M., Broggi M., Aquino D., Broggi G. (2013). Advanced 3-dimensional planning in neurosurgery. Neurosurgery.

[15] Javia L., Sardesai M.G. (2017). Physical Models and Virtual Reality Simulators in Otolaryngology. Otolaryngol. Clin. N. Am..

[16] Mamone V., Viglialoro R.M., Cutolo F., Cavallo F., Guadagni S., Ferrari V. (2017). Robust laparoscopic instruments tracking using colored strips. Lecture Notes in Computer Science (Including Subseries Lecture Notes in Artificial Intelligence and Lecture Notes in Bioinformatics).

[17] Pelagotti A., Del Mastio A., Uccheddu F., Remondino F. Automated multispectral texture mapping of 3D models. Proceedings of the 17th European Signal Processing Conference.

[18] Viergever M.A., Maintz J.B.A., Klein S., Murphy K., Staring M., Pluim J.P.W. (2016). A survey of medical image registration-under review. Med. Image Anal..

[19] Miao S., Wang Z.J., Liao R. (2016). A CNN Regression Approach for Real-Time 2D/3D Registration. IEEE Trans. Med. Imaging.

[20] Hajnal J.V., Hill D.L.G., Hawkes D.J. (2001). Medical Image Registration.

[21] Besl P.J., McKay N.D. (1992). A method for registration of 3-D shapes. IEEE Trans. Pattern Anal. Mach. Intell..

[22] Zhou Q.Y., Park J., Koltun V. (2016). Fast global registration. Lecture Notes in Computer Science (Including Subseries Lecture Notes in Artificial Intelligence and Lecture Notes in Bioinformatics).

[23] Chen D.-Y., Ouhyoung M. A 3D Model Alignment and Retrieval System. Proceedings of the International Computer Symposium, Workshop on Multimedia Technologies.

[24] Bücking T.M., Hill E.R., Robertson J.L., Maneas E., Plumb A.A., Nikitichev D.I. (2017). From medical imaging data to 3D printed anatomical models. PLoS ONE.

[25] Balafar M.A., Ramli A.R., Saripan M.I., Mashohor S. (2010). Review of brain MRI image segmentation methods. Artif. Intell. Rev..

[26] Buonamici F., Guariento L., Volpe Y. (2019). 3D Digital Surgical Planning: An Investigation of Low-Cost Software Tools for Concurrent Design. International Conference on Design, Simulation, Manufacturing: The Innovation Exchange.

[27] Ebel E., RTejournal T.S. (2014). Fabrication of FDM 3D objects with ABS and PLA and determination of their mechanical properties. Rapid Technol..

[28] Buonamici F., Carfagni M., Furferi R., Governi L., Saccardi M., Volpe Y. (2018). Optimizing fabrication outcome in low-cost FDM machines. Part 1-Metrics. Manuf. Technol..

[29] Witowski J.S., Pędziwiatr M., Major P., Budzyński A. (2017). Cost-effective, personalized, 3D-printed liver model for preoperative planning before laparoscopic liver hemihepatectomy for colorectal cancer metastases. Int. J. Comput. Assist. Radiol. Surg..

[30] Mussi E., Furferi R., Volpe Y., Facchini F., McGreevy K.S., Uccheddu F. (2019). Ear Reconstruction Simulation: From Handcrafting to 3D Printing. Bioengineering.

[31] Bootsma K., Dimbath E., Berberich J., Sparks J.L. (2017). Materials Used as Tissue Phantoms in Medical Simulation. Studies in Mechanobiology Tissue Engineering and Biomaterials.

[32] Kuo C.-C., Lai M.-Y. (2011). Development of an automatic vacuum degassing system and parameters optimization for degassing process. Indian J. Eng. Mater. Sci..

[33] Jiménez M., Romero L., Domínguez I.A., Espinosa M.D.M., Domínguez M. (2019). Additive Manufacturing Technologies: An Overview about 3D Printing Methods and Future Prospects. Complexity.

[34] Volpe Y., Furferi R., Governi L., Uccheddu F., Carfagni M., Mussa F., Scagnet M., Genitori L. (2018). Surgery of complex craniofacial defects: A single-step AM-based methodology. Comput. Methods Programs Biomed..

